# The Contribution of Yoga to the Psychosocial Rehabilitation and Social Reintegration of Incarcerated Individuals: A Systematic Review

**DOI:** 10.3390/healthcare14010070

**Published:** 2025-12-27

**Authors:** Konstantinos Georgiadis, Giorgos Tzigkounakis, Katerina Simati, Konstantinos Tasios, Ioannis Michopoulos, Vasileios Giannakidis, Athanasios Douzenis

**Affiliations:** 1 Department of Medicine, School of Health Sciences, National and Kapodistrian University of Athens, 106 79 Athens, Greece; georgiadesk@hotmail.com (K.G.);; 2 Department of Research, Health and Resilience Institute, 106 75 Athens, Greece; 3 2nd Department of Psychiatry—Forensic Psychiatry, Medical School, National and Kapodistrian University of Athens, Attikon Hospital, 124 62 Athens, Greece; 4 2nd Department of Psychiatry—Head, Eating Disorders Unit, Medical School, National and Kapodistrian University of Athens, Attikon Hospital, 124 62 Athens, Greece; 5 Special Centre for Mental Health of Prisoners, General Secretariat for Anti-Crime Policy, Ministry of Citizen Protection, 101 77 Athens, Greece

**Keywords:** yoga, incarceration, trauma recovery, emotional regulation, rehabilitation, correctional health, mindfulness, prison interventions

## Abstract

**Background/Objectives**: Incarcerated people experience high rates of trauma, psychological distress, and social marginalization. Yoga has been introduced in prisons as a trauma-sensitive mind–body practice, yet its rehabilitative contribution remains uncertain. This systematic review aimed to synthesize evidence on the feasibility and effectiveness of yoga interventions delivered in correctional settings. **Methods**: Following PRISMA guidelines and a preregistered PROSPERO protocol, we searched PubMed, PsycINFO, Cochrane CENTRAL, and Scopus for peer-reviewed publications from May 2012 to November 2025. Eligible studies involved structured yoga interventions for incarcerated populations and reported psychological, behavioral, or institutional outcomes. Two reviewers independently performed screening, data extraction, and quality appraisal using the Mixed-Methods Appraisal Tool (MMAT). **Results**: Ten studies reported in twelve publications and involving 1815 incarcerated individuals met the inclusion criteria. Interventions included Hatha-based protocols, *Krimyoga*, trauma-informed approaches, and multicomponent programs. Across randomized, quasi-experimental, and pre–post designs, yoga was feasible and acceptable. Reported benefits included reduced psychological distress, negative affect, anger, and trauma-related symptoms, as well as improved mood, self-regulation, and mindfulness. Evidence specific to women and girls was limited, but the available trauma-informed and gender-responsive studies suggested potential reductions in post-traumatic stress, depression, and anxiety, alongside increases in self-compassion. One large quasi-experimental cohort found lower reincarceration rates among yoga participants, although institutional outcomes were otherwise limited. Evidence was constrained by small samples, heterogeneous intervention formats, short follow-up, and variable outcome measures. **Conclusions**: Yoga appears to be a promising adjunct to rehabilitation in correctional settings. However, methodological limitations prevent firm conclusions. Larger, well-controlled studies with standardized outcomes and longer follow-up are needed to clarify effectiveness and support integration into correctional health and rehabilitation policy.

## 1. Introduction

Incarcerated individuals experience disproportionately elevated levels of psychological distress, caused not only by the loss of freedom but also often stemming from complex trauma, adverse childhood experiences (ACEs), structural inequalities, and cycles of social exclusion [1,2]. These vulnerabilities can predate incarceration and are further aggravated by the carceral environment. Prisons are frequently structured by intensified surveillance, rigid hierarchies, and interpersonal violence, factors that may exacerbate symptoms of anxiety, depression, and post-traumatic stress among incarcerated persons [3]. In such environments, institutional priorities often favor control and risk management over therapeutic intervention, which can entrench emotional dysregulation and reinforce behavior patterns that contribute to recidivism [4].

Multiple epidemiological studies confirm that incarcerated populations present with psychiatric disorders at rates significantly higher than the general public [5,6]. Moreover, young offenders, who represent about five percent of people in custodial settings, comprise a particularly vulnerable group within forensic psychiatric services [7]. This group faces substantial neurodevelopmental risk linked to early trauma and social marginalization, and they frequently present with emotional dysregulation and impaired executive functioning [8,9,10].

In this context, trauma can be understood as exposure to events involving actual or threatened harm that, in custodial populations, is often cumulative and associated with persistent difficulties in arousal regulation, emotion regulation, and behavioral control. Because these trauma-related sequelae are not purely cognitive, traditional rehabilitation programs such as cognitive behavioral therapy (CBT), talk therapy, or vocational training, although beneficial, often fall short of addressing the embodied impact of long-term incarceration and developmental trauma [11,12,13]. Trauma-informed correctional care frameworks integrate established evidence-based trauma treatments rather than replace them, and they emphasize helping incarcerated individuals develop safer coping strategies and increased emotional regulation within custodial settings [14,15].

Within this context, yoga has emerged as a promising embodied and trauma-sensitive intervention for incarcerated populations. Yoga is a physical and mental discipline that originated in India over 2000 years ago, and its name comes from the Sanskrit word *yuj*, meaning to join, reflecting the union of body, mind, and spirit [16]. Rooted in ancient Hindu philosophical traditions, contemporary yoga programs typically integrate physical postures (asana, Sanskrit: *āsana*), regulated breathing (pranayama, Sanskrit: *prāṇāyāma*), and meditative or contemplative practices, with the aim of cultivating attention, self-awareness, and behavioral restraint [17]. Theoretical models and empirical reviews describe yoga as a multicomponent mind–body practice that acts on both top-down cognitive control processes and bottom-up autonomic and interoceptive systems, thereby offering a plausible route to improved self-regulation in individuals with trauma-related distress.

Because trauma-related dysregulation is closely linked to altered autonomic and neurochemical functioning, neurobiological mechanisms provide an important bridge between embodied practices such as yoga and observed psychological change. On this basis, experimental evidence indicates that yoga can modulate key biological systems involved in stress and mood regulation. Magnetic resonance spectroscopy studies show that yoga practice increases cortical *γ*-aminobutyric acid (GABA) levels and that greater GABA increases are associated with reduced anxiety and improved mood [18,19]. Controlled studies measuring heart rate variability (HRV) also show that yoga practices can shift autonomic balance toward parasympathetic dominance, reflecting improved stress-regulation capacity [20,21,22,23]. Peripheral biomarker studies and meta-analytic findings also demonstrate that yoga and related mind–body practices can increase circulating brain-derived neurotrophic factor (BDNF) [24]. In addition, trials in adults with depression report decreases in serum cortisol following yoga interventions [25], and several systematic reviews conclude that yoga has clinically potent antidepressant effects [26,27,28,29]. Yoga is also associated with improvements in cardiovascular health [30,31,32,33], particularly relevant for incarcerated populations, who have disproportionately high rates of hypertension and cardiovascular risk [34,35].

Yoga has also demonstrated benefits for other conditions prevalent in custodial populations, such as attention-deficit/hyperactivity disorder (ADHD) [36]. ADHD is also highly overrepresented in correctional settings, with prevalence reaching about 30 percent in detained youth and around 25 percent in incarcerated adults [37,38], far above general population rates. These findings indicate that yoga-based approaches may hold particular relevance in prison contexts.

From a substance use perspective, yoga offers promising applications as a complementary intervention in addiction recovery and relapse prevention [39]. Several studies demonstrate its capacity to regulate craving, reduce relapse, and support emotional detoxification mechanisms, often targeted by mindfulness-based relapse prevention models [40,41,42]. Recent neurobiological perspectives suggest that yoga may modulate reward circuitry, stress systems, and inhibitory control, providing a mechanistic rationale for its application in addiction treatment [42,43].

Beyond these neurobiological, physiological, and clinical effects, yoga also cultivates introspection, self-discipline, and prosocial behaviors [44,45,46], which are crucial factors in the rehabilitation of individuals within high-risk correctional environments.

Yoga has gained increasing acceptance within correctional systems as a feasible and cost-efficient rehabilitative practice that can be delivered with minimal resources and high participant engagement. Evidence from randomized controlled trials in adult prisons indicates that yoga is well-tolerated, feasible to implement, and associated with improvements in impulse control, emotional stability, aggression attenuation, and reduced psychological distress [47,48]. Systematic reviews confirm that yoga and mindfulness programs in prisons yield small-to-moderate improvements in psychological well-being and behavioral functioning, supporting their acceptability and potential rehabilitative value in custodial environments [49,50,51,52]. Beyond adult populations, yoga has also been introduced in juvenile institutions as a complementary intervention designed to enhance attention, compliance, and treatment responsivity while reducing stress and supporting prosocial development, with early studies reporting positive engagement and strong feasibility in routine practice, although the evidence base in juvenile settings remains limited to a small number of pilot and single-site studies [53,54].

To clarify further why yoga may be uniquely relevant in custodial settings, it is helpful to distinguish between top-down and bottom-up regulation. Whereas top-down approaches rely primarily on cognitive control and reflective attention, bottom-up approaches act more directly on physiological and interoceptive processes via movement and breath [55]. In prison environments characterized by chronic stress and hyperarousal, where access to higher-order cognitive resources may be constrained, yoga, through controlled breathing (pranayama) and sustained postures and movement (asana), offers a predominantly bottom-up route that is mechanistically distinct from seated mindfulness practices. This is consistent with trauma-focused clinical literature describing bottom-up, sensory-awareness-based approaches as particularly useful for improving regulation when hyperarousal limits access to cognitive, top-down strategies [56]. A complementary explanatory model is the Theory of Constructed Emotion, which proposes that emotions are constructed as the brain categorizes interoceptive signals in context, and that changing bodily inputs can shape emotional experience, reactivity, and action tendencies [57,58].

Collectively, this evidence suggests that yoga is a practical, scalable, and well-accepted adjunct to existing correctional programming across both adult and youth custodial settings, supporting not only symptom reduction but also dignity and agency.

Given that existing findings are scattered across small heterogeneous studies, this systematic review evaluates the rehabilitative potential of yoga in custodial settings and its contribution to trauma recovery, behavioral control, and social reintegration.

## 2. Materials and Methods

### 2.1. Search Strategy

A structured search was conducted in PubMed, PsycINFO, Cochrane CENTRAL, and Scopus. All databases were searched from 1 January 2000 to 28 November 2025. In accordance with the preregistered PROSPERO protocol (CRD420251180905), we restricted eligibility to peer-reviewed studies with full texts published on or after 31 May 2012. The search strategy followed PRISMA 2020 guidance and adhered to the preregistered protocol in PROSPERO (CRD420251180905). Three conceptual domains were combined: yoga and mind–body practices, carceral settings, and psychological or rehabilitative outcomes. Controlled vocabulary terms were used where available, including MeSH terms in PubMed and Thesaurus headings in PsycINFO, and were paired with free-text keywords. Boolean operators were adapted for each database.

In PubMed, search terms included MeSH and TiAb variants for yoga, prisons, incarceration, rehabilitation, mental health, trauma, emotion regulation, substance use, aggression, prosocial behavior, and social reintegration. PsycINFO was searched using abstract fields for yoga, prison, incarceration, correctional facilities, and detention centers. Cochrane CENTRAL was searched using text words for yoga and custodial environments. Scopus searches used TITLE-ABS-KEY fields for yoga combined with incarceration-related terms and outcomes related to rehabilitation, mental health, trauma, emotional regulation, aggression, substance use, and prosocial behavior. All databases were searched for records published between 2000 and November 2025, but studies published before 31 May 2012 were excluded during screening in accordance with the prespecified eligibility criteria in the PROSPERO registration.

The full electronic search strings and the number of records retrieved from each database are provided in Supplementary Table S2, exactly as executed in the final search. All searches were limited to English-language, peer-reviewed publications. The final search was conducted on 28 November 2025. Grey literature, dissertations, theses, conference abstracts, commentaries, and non-peer-reviewed reports were excluded. Reference lists of all eligible studies and relevant reviews were screened manually to identify additional records.

### 2.2. Inclusion and Exclusion Criteria

Eligible studies included incarcerated adults or adolescents in prisons, correctional facilities, or detention settings. Interventions were required to use yoga as the primary modality, including asana-based yoga, pranayama, meditation-based yoga, or trauma-sensitive yoga, delivered for at least eight weeks, a duration chosen to allow meaningful psychological change and to reflect the typical length of yoga-based mental health trials [47,59,60,61]. Multicomponent programs were also included if yoga constituted a core component meeting this duration threshold. Eligible outcomes were psychosocial, emotional, behavioral, or neurophysiological markers relevant to rehabilitation or social reintegration. Study designs included randomized controlled trials, quasi-experimental studies, longitudinal designs, mixed-methods studies, and qualitative program evaluations.

Studies were excluded if yoga was combined with primarily pharmacological interventions and did not report psychosocial outcomes, if the intervention consisted solely of physical exercise without meditative or breathwork components, if the intervention was delivered for less than eight weeks, if the population involved correctional staff or community re-entry participants rather than individuals currently in custodial settings, or if no English full text or abstract was available.

### 2.3. Screening Process

Two reviewers independently screened all titles and abstracts, followed by full-text assessment of potentially eligible studies. Screening was conducted using Rayyan software (Rayyan Systems Inc., Doha, Qatar; available at https://www.rayyan.ai, accessed on 29 November 2025), which facilitated blinded review and study management. Disagreements were resolved through discussion and, when required, adjudication by a third reviewer.

### 2.4. Data Extraction and Appraisal

Data extraction followed a structured form that captured study setting, participant characteristics, intervention content and duration, instructor background, outcome domains, quantitative measures, qualitative themes, and implementation factors. Two reviewers extracted data independently and resolved discrepancies through discussion.

Methodological quality was assessed using the Mixed-Methods Appraisal Tool (MMAT 2018), applied according to each study’s design category. MMAT criteria were used to evaluate clarity of research questions, appropriateness of methodology, risk of bias, and coherence between data and conclusions. Calibration exercises were conducted before appraisal, and agreement was maintained through consensus procedures. The operationalization of MMAT items and the full appraisal table for all included studies are provided in Supplementary Table S1.

### 2.5. Theoretical and Analytical Framework

The synthesis was informed by three complementary frameworks. Polyvagal Theory provided a model for interpreting autonomic regulation and trauma recovery in custodial environments [62]. The Risk–Need–Responsivity model guided the interpretation of behavioral and rehabilitative effects in relation to criminogenic needs [63,64]. The Theory of Constructed Emotion was also used to interpret changes in emotional reactivity and behavioral control through the categorization of interoceptive signals in context [57,58].

These frameworks supported the interpretation of quantitative and qualitative findings and allowed the review to link intervention components to mechanisms relevant to rehabilitation.

### 2.6. Data Synthesis Strategy

Given the heterogeneity of study designs and outcomes, a narrative and convergent thematic synthesis was performed. Quantitative results and qualitative findings were integrated within shared domains that reflect rehabilitative processes. The synthesis focused on five areas: trauma recovery, emotional regulation, aggression and behavioral control, youth and gender-responsive rehabilitation, and institutional climate. Biomarker and physiological data were incorporated where available to support mechanistic interpretation. Contextual information on fidelity, feasibility, and implementation barriers was examined to understand real-world applicability in custodial settings.

## 3. Results

**Figure 1:**
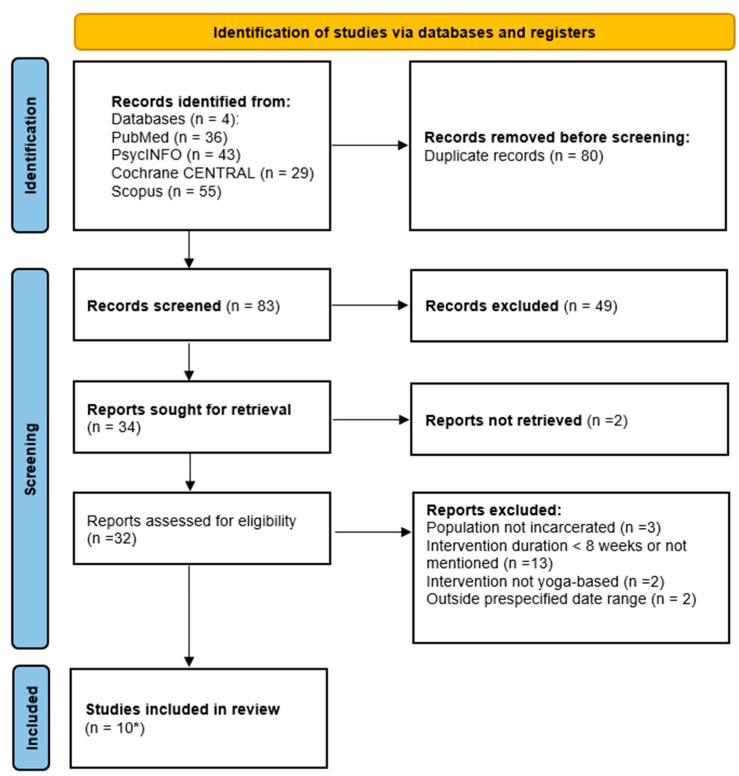
PRISMA 2020 flow diagram. * One RCT (Kerekes et al., 2017 [47]) had three eligible publications (Kerekes et al., 2017; Sfendla et al., 2018; Kerekes et al., 2019 [47,48,66]). These were treated as a single study in accordance with PRISMA guidance. Full search strategies and database results are provided in Supplementary Table S2.

A total of 163 records were identified through database searching. After the removal of 80 duplicates, 83 records were screened by title and abstract. Forty-nine were excluded at this stage. Thirty-four full texts were sought for retrieval, and two could not be obtained. Thirty-two full texts were assessed for eligibility. Following full-text screening, 22 reports were excluded for reasons related to population, duration, intervention type, or date range. Twelve publications met the criteria and represented ten unique studies, because three papers were secondary analyses of the same Swedish randomized controlled trial. The ten included studies involved a total of 1815 incarcerated participants (Figure 1).

### 3.1. Study Characteristics

The ten included studies were conducted in diverse correctional environments across the UK [65], Sweden [47,48,66], the United States [67,68,69,70,71], Israel [72], India [73], and Australia [74], involving adult or adolescent incarcerated individuals. Study designs included randomized controlled trials, quasi-experimental studies, and quantitative descriptive evaluations. Sample sizes ranged from small pilot cohorts to larger institutional programs. Key characteristics of the included studies are summarized in Table 1.

Intervention approaches included classical Hatha yoga [65], *Krimyoga* (a standardized prison-specific Hatha-based program) [47,48,66], trauma-informed yoga [68], yoga programs incorporating mindfulness components [69,70], expressive writing combined with yoga [67], anger-management-oriented yoga [69], and programs using the Indian Common Yoga Protocol [73].

Outcomes included psychosocial functioning, trauma-related symptoms, emotional regulation, aggression and behavioral control, prosocial behaviors, and institutional indicators such as disciplinary events. 

### 3.2. Psychological Distress, Mood, and General Psychosocial Functioning

Across the randomized controlled trials and the quasi-experimental cohort, yoga was consistently associated with improvements in psychological distress, mood, and related psychosocial indicators. In the UK RCT, participation in a 10-week standardized yoga program led to greater increases in positive affect and greater reductions in perceived stress and psychological distress than a wait-list control and was also linked with better behavioral control on a Go/No-Go task. In the Swedish *Krimyoga* RCT, both yoga and physical exercise groups improved, but the yoga group showed greater gains in emotional well-being and reductions in antisocial behavior, together with improved attention and impulse control. A secondary analysis of the UK prison yoga trial by Bilderbeck et al. [75] found that a higher number of yoga classes and frequent self-practice were associated with larger reductions in perceived stress and negative affect over time. Qualitative data from an Australian pilot program further described subjective improvements in mood, coping, and sense of calm among male prisoners who completed an 8-week yoga course.

### 3.3. Trauma-Related Symptoms, Mindfulness, and Resilience

Two studies explicitly targeted trauma and self-regulation among incarcerated women. In a mixed-methods trial in a US women’s state prison, a trauma-informed yoga and psychoeducation program was associated with reductions in post-traumatic stress, depression, and anxiety symptoms, as well as increases in self-compassion and perceived coping resources. In a pilot study with young women in a juvenile facility, an 8-week trauma-informed yoga and mindfulness intervention led to significant increases in mindful awareness, with self-report data indicating improved stress management and emotional self-regulation in the context of trauma histories. Resilience-related outcomes were examined in a small US wait-list RCT combining yoga with expressive writing. Compared with controls, participants in the intervention arm showed significant reductions in depressive symptoms and significant increases in resilience, emotional regulation, and self-esteem after the program, suggesting that yoga can be a useful component of broader psychosocial interventions aimed at strengthening adaptive functioning.

### 3.4. Emotional Regulation, Anger, and Aggression

Emotional regulation and anger-related outcomes were examined in several studies. A randomized wait-list–controlled trial of trauma-focused yoga for female inmates in two US prisons found improvements in perceived stress, depression, and self-awareness after 10 weeks. In the Australian qualitative pilot, participants reported feeling calmer, less reactive, and better able to pause before responding in conflict situations. Aggression was assessed quantitatively in the Swedish *Krimyoga* RCT and in a US pilot RCT of a Hatha-based anger-management program. The Swedish trial showed reduced aggression and antisocial behavior and better impulse-control task performance among yoga participants. In the US trial, feasibility was acceptable, and within-group decreases in anger, depression, and anxiety were observed in the yoga arm, with administrative records showing low infraction rates in both groups and no evidence of increased disciplinary problems among yoga participants.

### 3.5. Youth and Gender-Responsive Interventions

Youth-focused and gender-responsive interventions formed a smaller but important subset of the evidence. The trauma-informed yoga and mindfulness program for young incarcerated women showed that a manualized, developmentally adapted protocol is feasible in juvenile justice settings and can enhance mindful awareness and self-regulatory capacity. In the women’s state prison study, gender-specific, trauma-sensitive yoga emphasized bodily safety and was associated with increased bodily awareness, reduced hyperarousal, greater emotional safety, and improvements in post-traumatic stress disorder (PTSD), depressive symptoms, and self-compassion. Qualitative accounts from mixed-gender and all-male prisons also described benefits for emotional expression, self-control, and relational functioning, indicating that yoga can be adapted to different gendered and developmental needs within custodial settings.

### 3.6. Institutional and Post-Release Outcomes

Only a few studies assessed institutional or post-release indicators. The Israeli quasi-experimental cohort found no differences in short-term rearrests between yoga participants and matched controls but reported reductions in reincarceration over one to five years among those who attended yoga classes. Institutional data were examined in two studies. In the US anger-management RCT, administrative records showed low infraction rates in both yoga and control groups, with no evidence of increased disciplinary problems among yoga participants. Qualitative accounts from the Australian pilot also described calmer unit dynamics and fewer interpersonal conflicts, although these impressions were not quantitatively measured. Overall, available evidence suggests that yoga is institutionally safe and may support better post-release outcomes, though robust data on disciplinary events and recidivism remain limited.

## 4. Discussion

This systematic review synthesized evidence from ten studies with 1815 incarcerated adults and adolescents across diverse correctional settings. Yoga was generally feasible, acceptable, and associated with improvements in psychological distress, emotional regulation, trauma-related symptoms, aggression, and behavioral control, although the evidence is constrained by small samples and methodological heterogeneity. Across randomized controlled trials, yoga produced measurable benefits in stress, mood, and cognitive control. The UK RCT showed reductions in perceived stress and negative affect and improved behavioral inhibition on a Go/No-Go task, while the Swedish *Krimyoga* trial reported greater emotional well-being, reduced antisocial behavior, and better attention and impulse control than physical exercise. Overall, these findings suggest that yoga may enhance both affective stability and executive functioning in correctional environments.

Beyond these primary psychological outcomes, yoga-based interventions showed meaningful benefits for individuals with significant trauma histories. Trauma-informed programs delivered in women’s prisons produced reductions in post-traumatic stress, depression, and anxiety, alongside increases in self-compassion and perceived coping abilities. These patterns were echoed in a juvenile facility, where an adapted trauma-informed protocol improved mindful awareness and supported healthier responses to stress among young women.

These findings align with theoretical models that highlight the importance of embodied practices in restoring a sense of safety and in strengthening interoceptive awareness after chronic trauma exposure. From a polyvagal perspective, improvements in stress reactivity and emotional stability can be understood as shifts toward greater autonomic regulation and physiological safety following prolonged threat exposure [62]. From a correctional rehabilitation standpoint, these gains are also consistent with the responsivity principle of the Risk–Need–Responsivity model, whereby improved emotional regulation and self-control may enhance individuals’ capacity to engage with rehabilitative programming [64].

These findings can be further interpreted through the Theory of Constructed Emotion, which conceptualizes emotions as emergent brain-based constructions shaped by the ongoing categorization of interoceptive signals in context [57,58]. Within this framework, chronic stress and trauma bias emotional predictions toward threat, hyperarousal, and reactive behavioral responses. Yoga-based practices, by systematically modifying bodily states through breath regulation and movement, may alter interoceptive input and support the re-categorization of internal sensations, leading to reduced emotional reactivity and improved behavioral control. This bottom-up modulation offers a plausible explanatory pathway linking the observed reductions in stress, anger, and impulsivity to improved self-regulation in custodial settings, where sustained hyperarousal can otherwise constrain cognitive, top-down regulation.

In line with this mechanistic interpretation, programs specifically targeting anger and behavioral control produced complementary benefits. A US pilot randomized trial found that a Hatha-based intervention for anger self-management reduced anger, anxiety, and depression without increasing disciplinary incidents. The Swedish *Krimyoga* trial also reported reductions in aggression and antisocial behavior and noted improvements in impulse control relative to physical exercise. Qualitative accounts from Australia described calmer mood, greater patience, and improved ability to pause before reacting. These patterns indicate that yoga may enhance emotional regulation and behavioral restraint in custodial settings.

Furthermore, youth and gender-responsive adaptations showed that yoga can meet specific developmental and contextual needs. Trauma-informed programs for adolescent girls improved mindful awareness and stress responses, while interventions for adult women emphasized bodily safety and empowerment and were associated with greater self-compassion and reduced hyperarousal. These results align with trauma-informed correctional care frameworks that prioritize safety, choice, and non-coercive coping strategies.

Institutional and post-release outcomes, although infrequently assessed, were encouraging. The Israeli national cohort study found that yoga participation was linked to reduced reincarceration over five years, underscoring its potential value in lowering recidivism. Similar effects have been reported by other authors investigating mindfulness-based interventions and yoga in correctional settings, who likewise found reductions in recidivism among justice-involved populations [52,76,77,78]. A follow-up analysis of the UK study noted that inmates who continued personal practice after program completion reported lower stress and fewer negative perceptions [75].

These findings are consistent with earlier systematic reviews [49] and with pre-2012 studies [78,79,80,81,82] that reported reductions in anxiety, aggression, and impulse dysregulation and supported overall feasibility in correctional settings. Together, these results indicate a stable pattern of psychological and behavioral benefits across two decades of research.

Neurobiological evidence offers plausible mechanisms for these effects. Yoga practice has been shown to increase GABA [18,19], improve HRV [20,21,22,23], reduce cortisol [25,83,84], and elevate BDNF [83,85], all of which contribute to stress modulation and mood stability. An fMRI study of yoga meditation practitioners also found reduced right dorsolateral prefrontal cortex activation to negative emotional images compared with controls [86]. This pattern suggests that long-term yoga practice may support more efficient recruitment of frontal executive mechanisms during the regulation of emotional interference.

Despite promising findings, several methodological limitations remain. Many studies relied on small samples, lacked active controls, or used heterogeneous measures. Attendance was influenced by logistical constraints, and long-term behavioral outcomes were examined in only a few studies.

Future research should prioritize larger multisite trials, harmonized outcome domains, and longer follow-up to clarify institutional and reintegration effects. Current evidence indicates that yoga is a feasible and adaptable rehabilitative modality targeting emotion regulation, attention, and behavioral control, which may support safer institutional climates and reintegration, although more rigorous trials are still required.

## 5. Limitations

This review is limited by the small number of eligible studies, modest sample sizes, and heterogeneity in intervention formats and outcome measures. Several trials lacked active comparators, and long-term behavioral or post-release outcomes were examined in only a few studies. These factors restrict generalizability and highlight the need for larger, multisite, and rigorously controlled trials. A further limitation concerns potential self-selection bias. In most included studies, participation in yoga programs was voluntary, which may have produced systematic baseline differences between participants and non-participants, including differences in motivation, treatment expectations, and readiness for behavioral change. In parallel, evidence suggests that self-selected research participants can also show higher baseline vulnerability [87], which may contribute to larger apparent pre-to-post change in uncontrolled designs. Both patterns can limit representativeness and may bias observed effects, reducing external validity.

At the review level, only English-language, peer-reviewed studies were included, and grey literature, dissertations, and program reports were excluded, which may have introduced publication bias. The search was restricted to four major databases and to studies published from 2012 onward, which may have led to the omission of relevant earlier or non-indexed work. These factors limit generalizability and prevent meta-analysis, underscoring the need for larger and more standardized future research. Despite these constraints, the review’s strengths include adherence to a preregistered protocol, a comprehensive multi-database search, and rigorous dual screening and appraisal procedures.

## 6. Conclusions

Overall, yoga emerges as a feasible and adaptable rehabilitative modality in custodial settings, with convergent evidence for benefits in emotional regulation, behavioral control, and trauma-related symptoms. These changes are directly relevant to psychosocial rehabilitation aims, since improved self-regulation, stress management, and interpersonal functioning can support engagement with education, therapy, and vocational programs during incarceration. Although the evidence base remains limited, the alignment of psychological, behavioral, and emerging mechanistic findings supports yoga’s potential contribution to contemporary correctional rehabilitation. Continued research using more robust designs, larger samples, and extended follow-up is needed to clarify long-term effects on institutional behavior and post-release outcomes. For correctional services, these findings indicate that structured yoga programs can be integrated as low-cost adjuncts to existing psychological and educational interventions, provided they are implemented within a trauma-informed framework. From a public health perspective, even modest reductions in reoffending and reincarceration would have important implications for population mental health, community safety, and correctional costs. Future studies should therefore systematically monitor recidivism and other indicators of social reintegration, and identify which program components, delivery formats, and treatment durations are most closely associated with sustained behavioral change, social inclusion, and improved adjustment after release.

## Figures and Tables

**Table 1:** Study characteristics.

Study	Country	Population/Setting	Design	N (Total)	Intervention	Comparator	Outcomes
Bilderbeck et al., 2013 [65]	UK	Adult inmates, 7 prisons	RCT	100	10-week standardized yoga	Wait-list	Mood, stress, cognition
Danielly & Silverthorne, 2017 [71]	USA	Female inmates, 2 prisons	Wait-list RCT	50	10-week trauma-informed yoga	Wait-list	Stress, depression, anxiety, self-awareness, self-control
Kerekes et al., 2017 (incl. 2018, 2019) [47,48,66]	Sweden	Male and female inmates, 9 prisons	RCT	152	10-week *Krimyoga* (prison-specific Hatha yoga program)	Physical exercise	Affect, stress, sleep, impulsivity, aggression
Bartels et al., 2019 [74]	Australia	Male inmates, high-security prison	Pilot, quantitative + qualitative	8	8-week yoga program	None	Depression, anxiety, stress, self-esteem, affect, emotion regulation
Rousseau et al., 2019 [68]	USA	Women’s state prison	Mixed methods	12	8-week trauma-informed yoga (TIMbo) and psychoeducation	None	PTSD, depression, anxiety, self-compassion
Kovalsky et al., 2021 [72]	Israel	National prison cohort (matched)	Retrospective, quasi-experimental	1182	Weekly yoga classes	Matched non-yoga group	Recidivism (rearrest, reincarceration)
Nicotera et al., 2021 [70]	USA	Young women in a juvenile facility	Pilot, Pre–post	52	8-week trauma-informed yoga	None	Mindful awareness/attention (self-regulation)
Maity et al., 2025 [73]	India	Adult inmates, model jail	RCT (exploratory)	191	8-week common yoga protocol	Usual routine	Personality traits (S/R/T)
Ferdik et al., 2025 [67]	USA	Adult male inmates, medium-security prison	Wait-list RCT	28	8-week combined yoga + expressive writing	Wait-list	Depression, resilience, emotional regulation
Uebelacker et al., 2025 [69]	USA	Adult inmates, 2 facilities	Pilot RCT	40	10-week Hatha-based yoga targeting anger management	Health education	Anger, aggression, depression, anxiety, infractions

## Data Availability

No new data were created or analyzed in this study. Data sharing is not applicable to this article.

## Supplementary Materials

The following supporting information can be downloaded at https://www.mdpi.com/article/10.3390/healthcare14010070/s1, Table S1: MMAT 2018 appraisal of included studies; Table S2: databases and full electronic search strategies.
