# Supplementary material for: The Contribution of Yoga to the Psychosocial Rehabilitation and Social Reintegration of Incarcerated Individuals: A Systematic Review
Source: Healthcare (Basel). 2025 Dec 27;14(1):70. doi: 10.3390/healthcare14010070 (PMC12785455; doi:10.3390/healthcare14010070)
Supplement: Supplementary file 1 [file healthcare-14-00070-s001.zip › healthcare-4050596-supplementary.pdf]

Operationalization of MMAT (2018) for Randomized Controlled Trials

The methodological quality of randomized controlled trials (RCTs) was assessed with the Mixed Methods Appraisal Tool (MMAT, 2018). To ensure consistency and reproducibility, each MMAT item was operationalized with predefined decision rules, as follows.

Q1. Randomization appropriately performed

Randomization was rated "Yes" when the article explicitly described the study as randomized and reported a randomization method, for example computer generated sequence, random number table, or Excel randomization routine. It was rated "No" when allocation was clearly non random or when the paper claimed randomization but described a quasi systematic method such as alternation or allocation by availability. It was rated "Cannot tell" when randomization was mentioned but the method was not described in sufficient detail to judge its appropriateness.

Q2. Groups comparable at baseline

Baseline comparability was rated "Yes" when key baseline characteristics, such as age, sex, education, or baseline outcome scores, were presented for each group and no clinically meaningful or author-acknowledged imbalances were evident. It was rated "No" when there were clear baseline imbalances on one or more important variables. It was rated "Cannot tell" when baseline characteristics were not presented by group or when no between group comparison at baseline was reported.

Q3. Complete outcome data

Outcome data were rated "Yes" when overall attrition was 20 percent or less, or when higher attrition was fully explained, balanced between groups, and appropriately handled in the analysis, for example by imputation. The rating was "No" when attrition exceeded 20 percent and reasons for loss to follow up were unclear, differed between groups, or were not addressed analytically. It was rated "Cannot tell" when attrition or missing data were mentioned but not described in enough detail to judge their potential impact on study findings.

Q4. Blinding of outcome assessors

Blinding was rated "Yes" when the article explicitly stated that outcome assessors were blinded to group allocation, or when outcomes were obtained entirely through automated procedures that did not allow assessor influence. It was rated "No" when the article stated that assessors were not blinded or when blinding was clearly impossible and no steps were taken to minimise detection bias. It was rated "Cannot tell" when the paper did not provide any information on blinding of outcome assessment.

Q5. Adherence to the assigned intervention

Adherence was rated “Yes” when the study reported quantitative data on attendance or exposure, showed that approximately 70 percent of participants in the intervention group, or a clearly acceptable adherence level based on the study’s predefined criteria, completed the planned sessions, and documented some form of adherence monitoring, such as attendance logs or practice diaries. It was rated “No” when adherence was clearly poor, for example when completion rates were low, there was substantial non participation, or dropout was markedly higher in the intervention group. It was rated “Cannot tell” when adherence or attendance was only briefly mentioned, not quantified, or not monitored systematically.

For all items, when information in the publication was insufficient or ambiguous, the conservative choice “Cannot tell” was used. No attempts were made to contact study authors for clarification.

Table S1. MMAT 2018 Appraisal of Included Studies

Study	Design	Q1	Q2	Q3	Q4	Q5
Bilderbeck et al., 2013	Quantitative RCT	Yes	Yes	No	Can't tell	Can't tell
Danielly & Silverthorne, 2017	RCT	Can't tell	Can't tell	Yes	Can't tell	Can't tell
Kerekes et al., 2017	RCT	Can't tell	Yes	No	Can't tell	Can't tell
Sfendla et al., 2018*	Same dataset with Kerekes et al., 2017	Same dataset with Kerekes et al., 2017	Same dataset with Kerekes et al., 2017	Same dataset with Kerekes et al., 2017	Same dataset with Kerekes et al., 2017	Same dataset with Kerekes et al., 2017
Kerekes et al., 2019	Same dataset with Kerekes et al., 2017	Same dataset with Kerekes et al., 2017	Same dataset with Kerekes et al., 2017	Same dataset with Kerekes et al., 2017	Same dataset with Kerekes et al., 2017	Same dataset with Kerekes et al., 2017
Rousseau et al., 2019	Mixed-methods	No	No	Yes	Can't tell	Yes
Bartels et al., 2019	Quantitative descriptive	No	No	Yes	Can't tell	Yes
Kovalsky et al., 2021	Quantitative non-randomized	Yes	Yes	No	Yes	Can't tell
Nicotera & Viggiano, 2021	Quantitative descriptive	No	No	Yes	Can't tell	Yes
Maity et al., 2025	RCT	Yes	Yes	No	Can't tell	Can't tell
Ferdik et al., 2025	RCT	Yes	No	Yes	Can't tell	Can't tell
Uebelacker et al., 2025	RCT	Yes	Yes	Yes	No	Can't tell

*One RCT (Kerekes et al. 2017) had three eligible publications (Kerekes et al. 2017; Sfendla et al. 2018; Kerekes et al. 2019). These were treated as a single study in accordance with PRISMA guidance.

Table S2. Databases and Search Terms

Database	Results	Search Terms
PubMed	36	("Yoga"[Mesh] OR yoga[tiab] OR "trauma sensitive yoga"[tiab] OR "mind body interventions"[tiab] OR "mind-body intervention"[tiab] OR "mind body practice"[tiab])<br>AND<br>("Prisons"[Mesh] OR prison*[tiab] OR incarceration[tiab] OR incarcerated[tiab] OR "correctional facility"[tiab] OR "correctional facilities"[tiab] OR "juvenile detention"[tiab] OR "detention center"[tiab] OR "detention centres"[tiab])<br>AND<br>(rehabilitation[tiab] OR "rehabilitation"[Mesh] OR "mental health"[tiab] OR "mental health"[Mesh] OR "emotional regulation"[tiab] OR trauma[tiab] OR "stress disorders"[Mesh] OR aggression[tiab] OR "substance use"[tiab] OR addiction[tiab] OR "prosocial behavior"[tiab] OR "social reintegration"[tiab] OR "social adjustment"[Mesh])<br>AND<br>("2000/01/01"[Date - Publication] : "2025/03/31"[Date - Publication])
PsycINFO	43	Abstract: yoga AND Abstract: prison OR Abstract: incarcerated OR Abstract: incarceration OR Abstract: "correctional facility" OR Abstract: "detention center" AND Year: 2000 To 2025
Cochrane CENTRAL	29	yoga AND (prison OR incarcerated OR incarceration OR "correctional facility" OR detention)
Scopus	55	(TITLE-ABS-KEY (yoga)) AND (TITLE-ABS-KEY (prison OR incarceration OR incarcerated OR "correctional facility" OR "detention center")) AND (TITLE-ABS-KEY (rehabilitation OR "emotional regulation" OR "mental health" OR trauma OR aggression OR "substance use" OR addiction OR "prosocial behavior" OR "social reintegration")) AND (PUBYEAR > 1999 AND PUBYEAR < 2025)
